# An open-label, crossover study to compare different formulations and evaluate effect of food on pharmacokinetics of pimitespib in patients with advanced solid tumors

**DOI:** 10.1007/s10637-022-01285-9

**Published:** 2022-08-06

**Authors:** Yoshito Komatsu, Tsuneo Shimokawa, Kohei Akiyoshi, Masato Karayama, Akihiko Shimomura, Yasuyuki Kawamoto, Satoshi Yuki, Yuichi Tambo, Kazuo Kasahara

**Affiliations:** 1 Department of Cancer Center, Hokkaido University Hospital, Kita 14, Nishi 5, Kita-Ku, Sapporo, Hokkaido Japan; 2 Department of Respiratory Medicine, Yokohama Municipal Citizen’s Hospital, 1-1 Mitsuzawanishimachi, Kanagawa-ku, Yokohama, Kanagawa Japan; 3 Department of Medical Oncology, Osaka City General Hospital, 2-13-22 Miyakojima-hondori, Miyakojima-ku, Osaka, Japan; 4 Department of Chemotherapy, Hamamatsu University School of Medicine, 1-20-1 Handayama, Higashi-ku, Hamamatsu city, Shizuoka, Japan; 5 Department of Breast and Medical Oncology, National Center for Global Health and Medicine, 1-21-1 Toyama Shinjuku-ku, Tokyo, Japan; 6 Department of Gastroenterology and Hepatology, Hokkaido University Hospital, Kita 14, Nishi 5, Kita-ku, Sapporo, Hokkaido Japan; 7 Department of Respiratory Medicine, Kanazawa University Hospital, 13-1 Takara-machi, Kanazawa, Ishikawa Japan

**Keywords:** Crossover study, Effect of food, HSP90 inhibitor, Pharmacokinetics, Pimitespib, Advanced solid tumors

## Abstract

This study compared the bioavailability of two pimitespib formulations (Formulations A and B), evaluated the food effect on Formulation A, and evaluated the safety and efficacy of multiple pimitespib doses in patients with solid tumors. This clinical, pharmacological multicenter study had two cohorts and periods. A single dose of Formulation A or B was administered in a crossover design to compare the pharmacokinetics in Cohort 1. In Cohort 2, the effects of fed vs fasting conditions were evaluated among those receiving Formulation A. Subsequently, multiple Formulation A doses were administered to all patients for safety and efficacy assessments. In Cohorts 1 and 2, 12 and 16 patients, respectively, were analyzed for pharmacokinetics. Thirty patients were analyzed for safety and efficacy. Maximum concentration (C_max_), area under the curve (AUC)_last_, and AUC_inf_ geometric mean ratios for Formulations A and B (90% confidence interval [CI]) were 0.8078 (0.6569–0.9933), 0.7973 (0.6672–0.9529), and 0.8094 (0.6697–0.9782), respectively; 90% CIs were not within the bioequivalence range (0.80–1.25). In Cohort 2, mean C_max_, AUC_last_, and AUC_inf_ were higher in fed vs fasting conditions. No safety concerns emerged with single or multiple administration. Overall response rate, disease control rate, and median progression-free survival were 0%, 33%, and 1.5 months, respectively. Four patients had stable disease ≥ 5 months. Bioequivalence of the two formulations was unconfirmed. Systemic exposure of Formulation A was approximately 20% less than Formulation B. A high-fat/calorie meal increased the relative pharmacokinetics and bioavailability of a single 160-mg dose. Trial Registration: JapicCTI-184191 (Japan Pharmaceutical Information Center) registered on November 5, 2018.

**Supplementary Information:**

The online version contains supplementary material available at 10.1007/s10637-022-01285-9.

## Introduction

Pimitespib (TAS-116) is a novel orally active, selective heat shock protein 90 (HSP90) inhibitor currently under clinical development as an anticancer therapy. The main function of HSP90 is folding, stabilization, and activation of cellular “client” proteins such as KIT, PDGFRA, EGFR, and ALK, which contribute to protein homeostasis within cells [1]. Increased expression of HSP90 is linked to avoidance of apoptosis, increased proliferation [2], increased angiogenesis [3], and acquired resistance [4]; thus, high HSP90 levels are associated with poor prognosis and decreased survival in many cancer types [5–8]. The inhibition of HSP90 results in structurally unstable client proteins, which are degraded, consequently blocking the signal transduction system in cancer cells and leading to increased apoptosis and tumor death [9]. Therefore, HSP90 may be a potential therapeutic target, especially for advanced tumors presenting acquired resistance to approved agents, such as tyrosine kinase inhibitors.

The first-in-human phase I trial of pimitespib in patients with advanced solid tumors established a recommend dosage of 160 mg administered orally once daily in the empty stomach state for 5 consecutive days, followed by 2 days off per week per week, in a 21-day cycle. Preliminary efficacy was also observed; two patients with non-small cell lung cancer and one with gastrointestinal stromal tumor (GIST) achieved a partial response. The safety profile was acceptable; gastrointestinal disorders, creatinine increases, liver enzyme increases, and eye disorders were the most common treatment-related adverse events (TRAEs). These findings supported further clinical development of pimitespib [10].

A phase II study of pimitespib was conducted in patients with advanced GIST who had failed or were intolerant to imatinib, sunitinib, and regorafenib treatments. In this refractory population, pimitespib demonstrated significant activity, with a median progression-free survival (PFS) of 4.4 months (95% CI 2.8 − 6.0) [11]. Gastrointestinal disorders and increased serum creatinine were commonly observed TRAEs.

The phase III CHAPTER-GIST-301 trial [12] found that pimitespib significantly increased the median PFS of patients with advanced GIST refractory or intolerant to treatment with imatinib, sunitinib, and regorafenib. Median PFS was 2.8 months (95% CI: 1.6–2.9) for pimitespib vs. 1.4 months (95% CI: 0.9–1.8) for placebo. The hazard ratio (HR) for PFS was 0.51 (95% CI: 0.30–0.87) (p = 0.006, stratified log-rank test). The safety profiles were similar to phase 1 and phase 2 studies. Based on the promising results obtained in this and previous trials, the clinical development of pimitespib is ongoing.

Clinical trials of pimitespib have utilized one of two formulations (Formulation A, pimitespib 40 mg × 4; Formulation B, pimitespib 10 mg × 1 and 50 mg × 3). Formulation A was used in a phase III (patients with GIST) study [12]. Formulation B was used in phase I (patients with solid tumors) and phase II (patients with GIST) studies [10, 11]. Therefore, it is necessary to compare the pharmacokinetics (PK) profiles of pimitespib from Formulation A with Formulation B. The new 40 mg strength Formulation A was developed to allow for more convenient drug administration because the administration of pimitespib starts at 160 mg/patient. Additionally, Formulation A development aimed to achieve a smaller pill for easier intake.

Because pimitespib is administered orally, it is necessary to evaluate its PK under the effect of food [13, 14]. Administration of a drug with food could impact the drug’s absorption [15]. Food may affect the gastrointestinal pH [16], emptying, and motility [17]. The macronutrient profile of the meal may also affect drug absorption. A meal high in fat may increase the bioavailability of lipophilic drugs [17].

The primary objective of this study was to compare the PK parameters between pimitespib Formulations A and B and compare the PK parameters of Formulation A under fasting and fed conditions in patients with advanced malignant tumors, including malignant soft tissue tumors or stromal tumors, refractory to conventional therapy or without standard therapy available. The secondary objective was to evaluate the safety and efficacy of multiple doses of pimitespib during the consecutive administration period.

## Materials and methods

### Patients

Key inclusion criteria were ≥ 20 years of age, histologically confirmed solid tumor(s), Eastern Cooperative Group Performance Status (ECOG PS) score of 0–1, adequate organ function, and the ability to take medications orally and adequately eat meals (i.e., without a feeding tube). Key exclusion criteria were corrected visual acuity of < 0.5 (using the International Visual Acuity Measurement Standard) for both eyes, gastrointestinal dysfunction (e.g., history of gastrectomy, including partial gastrectomy) that may markedly interfere with the absorption of pimitespib, or undergoing treatment or taking any prohibited medication or food that has a strong or moderate inhibitor effect, or a strong or moderate inducing effect of cytochrome P450 3A within 7 days before the scheduled study drug administration day. All patients provided informed consent before study participation.

### Study design

This clinical, pharmacological, multicenter study was conducted in Japan and consisted of two cohorts (Cohorts 1 and 2) and two periods. The patients were first assigned to the pharmacokinetic evaluation period to investigate PK parameters of each formulation (Cohort 1) or the effect of food (Cohort 2), and then to the consecutive administration period (Supplementary Fig. 1).

In both cohorts, a randomized cross-over design was used. In Cohort 1, during the PK evaluation period, pimitespib was administered under fasting conditions as a single administration of Formulation A (40 mg × 4) followed by a single dose of Formulation B (10 mg × 1 and 50 mg × 3), or a single dose of Formulation B followed by a single dose of Formulation A.

Patient enrollment in Cohort 2 was initiated after enrollment of Cohort 1 was completed. In Cohort 2, a single dose of Formulation A (40 mg × 4) was administered first under fed and then fasting conditions or first under fasting and then fed conditions.

In both cohorts, after the PK evaluation period, patients who met all the criteria for the continuation were transferred to the consecutive administration period. For the consecutive administration period, Formulation A was administered on an empty stomach (at least 1 h before or 2 h after eating) for 5 days, followed by a 2-day rest period per week.

For fasting conditions, patients were required to fast at least 10 h before dosing and at least 4 h after dosing and abstain from drinking water 1 h before and after administration, excluding the water consumed at the time of dosing. For fed conditions, patients were required to fast for at least 10 h before dosing and at least 4 h after dosing (except for the scheduled study meal) and abstain from drinking for 1 h before and after administration, except for drinking water. The study drug was administered within 30 min of completing the meal. The study meal was a high-fat (approximately 50% of the total caloric content of the meal) and high-calorie (572–715 kcal) meal considering the weight ratio of Japanese to American individuals. The meal’s nutritional value was adjusted based on the body weight of Japanese patients according to the US FDA standard guidance. It was recommended that study drug dosing be done with 100–200 (usually 150) mL of water.

The institutional review board approved the study protocol at each study site. This study was conducted in compliance with the ethical principles in the Declaration of Helsinki, Good Clinical Practice (GCP), International Council for Harmonisation GCP, and all local regulatory requirements.

### Study outcomes

The primary PK outcome included the following parameters for Formulations A and B administered under fasting conditions in Cohort 1, and Formulation A administered under fasting and fed conditions in Cohort 2 during the PK evaluation period: maximum observed plasma concentration (C_max_), area under the plasma concentration–time curve from time 0 to the time of the last measurable plasma concentration (AUC_last_), and area under the plasma concentration–time curve from time 0 to infinity (AUC_inf_) in the PK evaluable population.

Secondary outcomes were safety, as measured by adverse events (AEs) and TRAEs, and efficacy, which included overall response rate (ORR), disease control rate (DCR), and PFS in the efficacy evaluable population. ORR was defined as the proportion of patients with the best overall response of complete response (CR) or partial response (PR). DCR was defined as the proportion of patients with the best overall response of CR, PR, stable disease (SD), or non-CR/non-progressive disease (PD). PFS was defined as the time from enrollment to PD or death from any cause, whichever occurred first. Response was determined according to the RECIST criteria (version 1.1). Reported AEs were graded according to the Common Terminology Criteria for Adverse Events version 4.03 for severity.

Patients underwent hematologic, coagulation, biochemical laboratory examinations, urinalysis, electrocardiogram, ophthalmologic examination, and vital sign and body weight assessments. Blood collection occurred before pimitespib administration and at 0.5, 1, 2, 4, 6, 8, 10, 24, and 48 h after administration at the first and second doses.

### Statistical analysis

The sample size was based on the guidelines provided in the following two publications from the US Food and Drug Administration: Food-Effect Bioavailability and Fed Bioequivalence Studies and Statistical Approaches to Establishing Bioequivalence [13, 14]. A total of 12 patients were planned to be assigned to each cohort. The enrolled population included all patients who were enrolled in the study. The treated population included all patients in the enrolled population who had received at least one dose of the study drug. The PK evaluable population included all patients in the treated population who had the blood collection timepoints necessary to calculate the PK parameters. The efficacy evaluable population included all patients who had at least one tumor evaluation after the initial study drug administration.

For Cohort 1 analyses, the values of the natural log-transformed PK parameters (C_max_, AUC_last_, AUC_inf,_ terminal elimination rate constant [λz], and mean residence time) were analyzed using analysis of variance (ANOVA), using Phoenix^®^ WinNonlin^®^ Ver. 8.1. The ANOVA model included treatment (Formulation A versus Formulation B), treatment period, and treatment sequence as fixed effects, and patients nested within treatment sequence as a random effect. The geometric mean ratio (GMR) and 90% confidence interval (CI) of Formulation A to B were calculated from the model. Formulations A and B were considered of comparable bioavailability if the 90% CIs for the GMR of PK parameters (C_max_, AUC_last_, and AUC_inf_) between the two treatments were within the equivalence range limits of 0.80 to 1.25. The time to maximum plasma concentration (t_max_) was not transformed and was analyzed using Wilcoxon’s signed-rank test, conducted using EXSUS version 10.0.3. The significance level was set at 5%.

For the analyses related to Cohort 2, the ANOVA model included treatment (fasting and fed conditions), treatment period, and treatment sequence as fixed effects, and patients nested within treatment sequence as a random effect. The GMR and 90% CI of the fed condition to the fasting condition were calculated. The absence of a food effect was to be concluded if the 90% CI for the GMR of PK parameters (C_max_, AUC_last_, and AUC_inf_) between the two treatment conditions (fasting versus fed) was within the equivalence range limits of 0.80 to 1.25.

PFS curves were prepared, and point estimates with 95% CI for the median PFS were calculated using the Kaplan–Meier method. SAS Version 9.4 (SAS Institute Inc., Cary, NC, USA) and SAS/STAT 14.2 were used for statistical processing.

## Results

### Patient characteristics

The first patient was enrolled in January 2019, and the last patient’s final observation was in November 2020. In Cohort 1, 13 patients received the study drug Formulations A and B. One patient was excluded from the PK analysis because of issues with the storage of the blood samples. Thus, 12 patients were included in the PK evaluable population. In Cohort 2, 17 patients received Formulation A under fasting and fed conditions. One patient was excluded from the PK analysis because of insufficient meal consumption. Thus, 16 patients were included in the PK evaluable population (Supplementary Fig. 2).

**Fig. 1:**
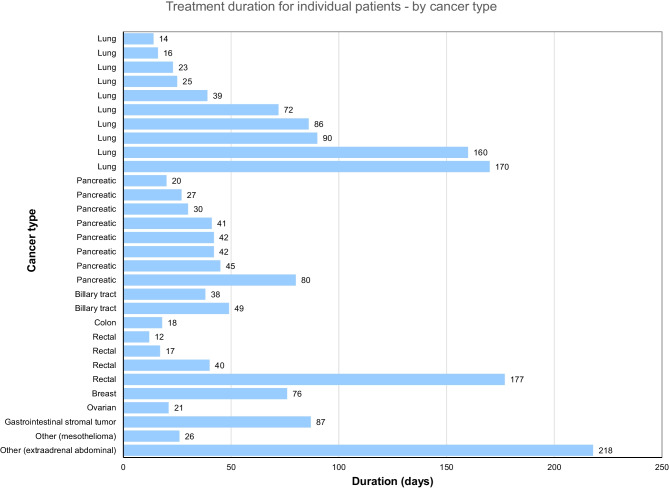
Treatment duration in days for individual patients by cancer type

In Cohort 1, the median (range) age was 64.0 (38–74) years, 61.5% of patients were male, and 61.5% had an ECOG PS of 0. In Cohort 2, the median (range) age was 59.0 (40–78) years, 52.9% were male, and 35.3% had an ECOG PS of 0. The most common tumor types were lung cancer (33.3%, *n* = 10), followed by pancreatic cancer (26.7%, *n* = 8) and rectum (13.3%) and biliary tract (6.7%) cancers (Table 1). All patients (*n* = 30) treated in the PK evaluation period proceeded to the consecutive administration period. During the consecutive administration period, median treatment duration was 20 days (interquartile range 11.0–46.0), and the relative dose intensity was 87.1% (interquartile range 57.5–100.0). The longest treatment duration was 218 days for a patient with an extra-adrenal abdominal paraganglioma, followed by 177 days for a patient with rectal cancer and 170 and 160 days for two patients with lung cancers (Fig. 1).

**Table 1:** Patient demographics and baseline characteristics

	Cohort 1	Cohort 2	Total
	*n* = 13	*n* = 17	*N* = 30
Sex			
Male	8 (61.5)	9 (52.9)	17 (56.7)
Female	5 (38.5)	8 (47.1)	13 (43.3)
Age (years)			
Mean (standard deviation)	62.2 (11.1)	59.6 (12.0)	60.7 (11.5)
Median (min–max)	64.0 (38–74)	59.0 (40–78)	63.0 (38–78)
Age Category 1 (years)			
< 65	7 (53.8)	10 (58.8)	17 (56.7)
≥ 65	6 (46.2)	7 (41.2)	13 (43.3)
Age Category 2 (years)			
< 40	1 (7.7)	0	1 (3.3)
40–<50	1 (7.7)	4 (23.5)	5 (16.7)
50–<60	2 (15.4)	5 (29.4)	7 (23.3)
60–<70	5 (38.5)	3 (17.6)	8 (26.7)
≥ 70	4 (30.8)	5 (29.4)	9 (30.0)
Height (cm)			
Mean (standard deviation)	163.21 (6.83)	162.94 (9.50)	163.05 (8.31)
Median (min–max)	166.90 (151.0–171.7)	158.50 (152.7–184.6)	161.50 (151.0–184.6)
Weight (kg)			
Mean (standard deviation)	58.36 (12.63)	59.63 (11.60)	59.08 (11.86)
Median (min–max)	54.70 (37.4–82.6)	58.40 (43.0–79.9)	56.10 (37.4–82.6)
ECOG PS			
0	8 (61.5)	6 (35.3)	14 (46.7)
1	5 (38.5)	11 (64.7)	16 (53.3)
Race			
Asian	13 (100.0)	17 (100.0)	30 (100.0)
Cancer type of primary tumor			
Biliary tract	1 (7.7)	1 (5.9)	2 (6.7)
Breast	0	1 (5.9)	1 (3.3)
Colon	1 (7.7)	0	1 (3.3)
Gastrointestinal stromal tumor	0	1 (5.9)	1 (3.3)
Lung	3 (23.1)	7 (41.2)	10 (33.3)
Ovary	0	1 (5.9)	1 (3.3)
Pancreas	6 (46.2)	2 (11.8)	8 (26.7)
Rectum	2 (15.4)	2 (11.8)	4 (13.3)
Other	0	2 (11.8)	2 (6.7)

Data are *n* (%), unless stated otherwise

*ECOG PS* Eastern Cooperative Oncology Group Performance Status

### Study endpoints

#### Primary outcome: PK analysis of Cohorts 1 and 2

**Fig. 2:**
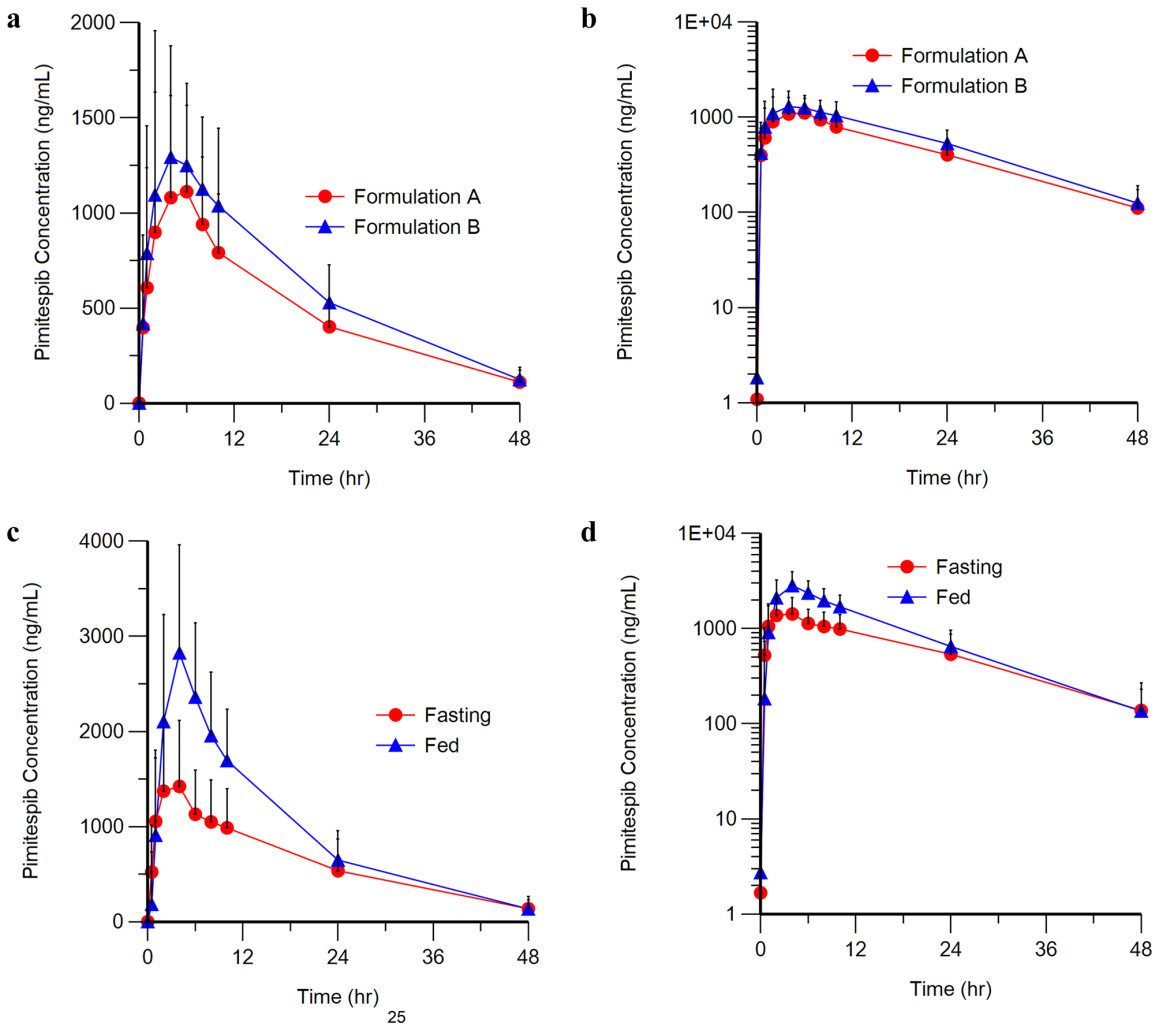
Mean + standard deviation plasma concentration–time profiles of pimitespib after administration **a** Formulations A and B (Cohort 1), linear scale, *n* = 12, **b** Formulations A and B (Cohort 1), logarithmic scale, *n* = 12, **c** Formulation A under fasting and fed conditions (Cohort 2), linear scale, *n* = 16, **d** Formulation A under fasting and fed conditions (Cohort 2), logarithmic scale, *n* = 16

Figure 2 shows the mean plasma concentration–time profiles of Formulations A and B under fasting conditions and Formulation A under fasting and fed conditions. In Cohort 1, the mean C_max_, AUC_last_, and AUC_inf_ with Formulation B were 1519 ng/mL, 29538 ng·h/mL, and 31933 ng·h/mL, respectively and with Formulation A, 1237 ng/mL, 23511 ng·h/mL, and 25192 ng·h/mL, respectively.

The GMRs for Formulations A and B were C_max_: 0.8078 (90% CI 0.6569–0.9933), AUC_last_: 0.7973 (90% CI 0.6672–0.9529), and AUC_inf_: 0.8094 (90% CI 0.6697–0.9782) (Table 2). As the 90% CIs of these PK parameters were not within the range of 0.80–1.25, Formulations A and B did not meet the bioequivalence criteria. The systemic exposure with Formulation A was approximately 20% less than Formulation B. The intra-subject coefficient of variation (CV%) for C_max_, AUC_last_, and AUC_inf_ was 28.5%, 24.4%, and 24.9%, respectively. The respective inter-subject variability (CV%) was 63.8%, 52.6%, and 54.4%. The t_max_ for Formulations A (*n* = 12) and B (*n* = 12) were (median [range]) 4.04 (1.92–7.68) h and 4.88 (1.88–7.75) h, *p* = 0.4131. The mean half-life (t_1/2_) was similar between Formulation A and Formulation B (12.85 h and 12.55 h, respectively).

**Table 2:** GMRs and the corresponding 90% CIs for pharmacokinetic parameters of Formulations A and B under fasting conditions (Cohort 1) and Formulation A under fed and fasting conditions (Cohort 2)

Cohort 1 (Formulation A/Formulation B)			
	GMR	90% CI	
		Lower	Upper
C_max_	0.8078	0.6569	0.9933
AUC_last_	0.7973	0.6672	0.9529
AUC_inf_	0.8094	0.6697	0.9782
λz	0.9672	0.8452	1.1069
MRT	1.0203	0.9060	1.1490

Cohort 2 (Fed/Fasting)			
	GMR	90% CI	
		Lower	Upper
C_max_	1.9206	1.5775	2.3384
AUC_last_	1.5668	1.3654	1.7978
AUC_inf_	1.6399	1.4520	1.8522

*AUC*_*inf*_ area under the plasma concentration–time curve from time 0 to time infinity, *AUC*_*last*_ area under the plasma concentration–time curve from time 0 to the time of the last measurable plasma concentration, *CI* confidence interval, *C*_*max*_ maximum observed plasma concentration, *GMR* geometric mean ratio, *MRT* mean residence time, *λz* terminal elimination rate constant

In Cohort 2, the mean C_max_, AUC_last_, and AUC_inf_ were 3046 ng/mL, 45479 ng·h/mL, and 49345 ng·h/mL, respectively, under fed conditions compared with 1625 ng/mL, 29922 ng·h/mL, and 29384 ng·h/mL, respectively, under fasting conditions. The variability for C_max_ and AUC_last_ under fed conditions (31.7% and 33.8%) was slightly lower compared with that for fasting conditions (43.5% and 46.8%). The CV% for AUC_inf_ was similar under fasting (35.4%) and fed (32.6%) conditions.

The GMRs (90% CIs) of Formulation A under fasting and fed conditions were C_max_: 1.9206 (1.5775–2.3384), AUC_last_: 1.5668 (1.3654–1.7978), and AUC_inf_: 1.6399 (1.4520–1.8522) (Table 2). The 90% CIs of C_max_, AUC_last_, and AUC_inf_ were not within the range of 0.80–1.25. The C_max_, AUC_last_, and AUC_inf_ were not statistically comparable under fasting and fed conditions. The t_max_ (90% CIs) for Formulation A under fasting conditions (*n* = 16) was 2.03 (0.93–7.53) and that for fed conditions (*n* = 16) was 4.02 (1.07–10.03), *p* = 0.0490. The mean t_1/2_ was similar under fasting and fed conditions (12.54 h and 10.02 h, respectively).

#### Secondary outcomes: safety and efficacy

Safety findings in the PK evaluation period are summarized in Supplementary Table 1. In the consecutive administration period, 27 patients (90.0%) experienced an AE, and 14 patients (46.7%) had Grade 3 or higher AEs (Table 3).

**Table 3:** Adverse events with an incidence ≥ 10% during the consecutive administration period

	Total (*N* = 30)				
	All Grade	Grade 3	Grade 4	Grade 5	Grade ≥ 3
Any events	27 (90.0)	13 (43.3)	0	1 (3.3)	14 (46.7)
Diarrhea	17 (56.7)	3 (10.0)	0	0	3 (10.0)
Nausea	10 (33.3)	0	0	0	0
Weight decreased	10 (33.3)	0	0	0	0
Decreased appetite	8 (26.7)	1 (3.3)	0	0	1 (3.3)
Aspartate aminotransferase increased	6 (20.0)	1 (3.3)	0	0	1 (3.3)
Alanine aminotransferase increased	5 (16.7)	1 (3.3)	0	0	1 (3.3)
Malaise	5 (16.7)	1 (3.3)	0	0	1 (3.3)
Pyrexia	5 (16.7)	0	0	0	0
Vomiting	5 (16.7)	0	0	0	0
Anemia	4 (13.3)	4 (13.3)	0	0	4 (13.3)
Blood alkaline phosphatase increased	4 (13.3)	2 (6.7)	0	0	2 (6.7)
Proteinuria	4 (13.3)	1 (3.3)	0	0	1 (3.3)
Hyperkalemia	3 (10.0)	1 (3.3)	0	0	1 (3.3)
Hypoalbuminemia	3 (10.0)	0	0	0	0

Data are *n* (%)

In the consecutive administration period, 83.3% (25/30) of patients experienced TRAEs, and 33.3% (10/30) had Grade 3 or higher TRAEs. TRAEs with an incidence of ≥ 15% included diarrhea (53.3%), decreased appetite (23.3%), nausea (20.0%), and malaise (16.7%). Grade 3 or higher TRAEs with an incidence of ≥ 10% were anemia (13.3%) and diarrhea (10.0%). No TRAEs led to death or treatment discontinuation. One patient died from disease progression (not considered a TRAE). Other serious TRAEs observed were anemia (two patients) and gastrointestinal hemorrhage (one patient).

During the consecutive administration period, the ORR was 0%. 10 patients (33.3%) had SD; five with lung cancer, and one patient each with rectal cancer, GIST, pancreatic cancer, breast cancer, and extra-adrenal abdominal paraganglioma. Twenty patients (66.7%) had PD. The DCR was 33.3% (95% CI 17.3–52.8), and median PFS was 1.5 months (95% CI 1.3–1.7).

## Discussion

The primary objectives of this study were to compare the bioavailability of pimitespib Formulations A and B and to evaluate the effect of food on the bioavailability of Formulation A. Secondarily, we also assessed the safety and anti-tumor efficacy of multiple dosing of pimitespib 160 mg/day orally in patients with malignant tumors, including malignant soft tissue tumors or stromal tumors, that were refractory to conventional therapy. During the PK evaluation period, patients in Cohort 1 receiving Formulation B had a higher mean C_max_ (1519 and 1237 ng/mL), AUC_last_ (29538 and 23511 ng·h/mL), and AUC_inf_ (31933 and 25192 ng·h/mL) compared with those receiving Formulation A; thus, the results indicate that pimitespib Formulations A and B did not fulfill the bioequivalence criteria. The variability among patients in this study was high. The intra-subject CV% for C_max_, AUC_last_, and AUC_inf_ were 28.5%, 24.4%, and 24.9%, respectively, and the respective values for inter-subject CV% were 63.8%, 52.6%, and 54.4%. In Cohort 2, the mean C_max_, AUC_last_, and AUC_inf_ were higher under fed conditions than fasting conditions. Of note, there was no significant difference in t_max_ between the two formulations. Furthermore, there were differences in systemic exposure, with nearly 20% greater exposure to Formulation B than Formulation A. Given the sizeable variability among patients, further investigation is needed to confirm the present results, with a larger sample and higher statistical power.

The C_max_ and AUC under fed conditions were approximately 1.9- and 1.6-fold higher, respectively, than those under fasting conditions. It was considered that the bioavailability of pimitespib was increased due to food intake, which leads to increased stomach acid secretion and increased blood concentration. Pimitespib solubility in the gastrointestinal tract increased under fed conditions. The t_max_ of Formulation A was significantly longer under fed conditions than fasting conditions. These findings should be considered when establishing the dosing instructions for pimitespib.

The safety profile between fed and fasting states and Formulations A and B in the PK evaluable period were comparable. During the consecutive administration period, the safety profile remained consistent with previous studies [11]. Moreover, no new safety concerns were identified; 67% of TRAEs were Grade 1 or 2 in severity, and the study treatment was manageable compared with other HSP90 inhibitors [18–20].

The patients in our study had an ORR of 0%, a DCR of 33%, and a median PFS of 1.5 months. Twenty patients experienced PD, and 10 had SD. In a phase III (patients with GIST) study using Formulation A, pimitespib significantly increased the median PFS [12].

Four patients, two with lung cancer, one with rectal cancer, and one with extra-adrenal abdominal paraganglioma, were treated for more than 5 months. One patient with lung cancer had an *EGFR* (an HSP90 client protein) mutation, while the other did not have any detectable mutation in HSP90 client proteins. The patient with rectal cancer had a *K-ras* mutation, and whether the patient with extra-adrenal abdominal paraganglioma had mutations was unknown. However, most hereditary paraganglioma patients have *VHL*, *NF1*, *SDHD*, *SDHAF2*, *SDHC*, *SDHB*, *SDHA*, *TMEM127*, or *MAX* gene mutations [21]. *RET* and *VHL* are HSP90 clients [22], and it has been reported that the expression of *HIF-1α* and *HIF-2α* (an HSP90 client) were implicated in the pathogenesis of paraganglioma with SDHB and SDHD mutations [23]. Therefore, inhibition of HSP90 might contribute to long-term SD in patients with extra-adrenal abdominal paraganglioma.

This study had two important limitations. First, the generalizability of the study is limited as the population was entirely Asian (only Japanese patients were enrolled). Second, this study included a small sample, and the inter-individual variability was quite high. The sample size was determined to be 12 patients according to the statistical guidance for establishing bioequivalence [13] and the guidance provided by the US Food and Drug Administration for food-effect bioavailability and fed bioequivalence studies [14] in patients with solid cancer. Based on the findings of our study, we expect that a larger sample size and higher statistical power would allow for improved evaluation in PK studies in cancer patients.

In conclusion, the systemic exposure of Formulation A was 20% lower than that of Formulation B. However, this difference did not seem to have a significant clinical impact on the efficacy and safety of pimitespib. The safety profile of pimitespib Formulation A in this study was tolerable, manageable, and consistent with previous studies. To understand the bioequivalence between these two formulations, further investigation via population PK studies is needed. A high-fat and high-calorie meal affected the PK of a single dose of 160 mg pimitespib, with increased relative bioavailability and delayed t_max_. Therefore, the administration of pimitespib on an empty stomach as recommended and implemented is reasonable.

## Supplementary Information

Below is the link to the electronic supplementary material.

Supplementary file1 (DOCX 251 KB)

## Data Availability

The study data will not be shared according to the Sponsor’s policy on data sharing, which can be found at https://www.taiho.co.jp/en/science/policy/clinical_trial_information_disclosure_policy/.

## Abbreviations

AE: Adverse event
ANOVA: Analysis of variance
AUC_inf_: Area under the plasma concentration–time curve from time 0 to infinity
AUC_last_: Area under the plasma concentration–time curve from time 0 to the time of the last measurable plasma concentration
CI: Confidence interval
C_max_: Maximum observed plasma concentration
CR: Complete response
CV%: Coefficient of variation
DCR: Disease control rate
ECOG PS: Eastern Cooperative Oncology Group Performance Status
GCP: Good Clinical Practice
GIST: Gastrointestinal stromal tumor
GMR: Geometric mean ratio
HSP90: Heat shock protein 90
MRT: Mean residence time
ORR: Overall response rate
PD: Progressive disease
PFS: Progression-free survival
PK: Pharmacokinetics
PR: Partial response
SD: Stable disease
λz: Terminal elimination rate constant
t_max_: Time to maximum plasma concentration
TRAE: Treatment-related adverse event
t_1/2_: Half-life.
