# Supplementary material for: An open-label, crossover study to compare different formulations and evaluate effect of food on pharmacokinetics of pimitespib in patients with advanced solid tumors
Source: Invest New Drugs. 2022 Aug 6;40(5):1011–20. doi: 10.1007/s10637-022-01285-9 (PMC9395490; doi:10.1007/s10637-022-01285-9)

**An open-label, crossover study to compare different formulations and evaluate effect of food on pharmacokinetics of pimitespib in patients with advanced solid tumors**

**Journal name:** Investigational New Drug

Yoshito Komatsu, MD^1^, Tsuneo Shimokawa, MD^2^, Kohei Akiyoshi, MD^3^, Masato Karayama, MD^4^, Akihiko Shimomura, MD^5^, Yasuyuki Kawamoto, MD^1^, Satoshi Yuki, MD^6^, Yuichi Tambo, MD^7^, Kazuo Kasahara, MD^7^

^1^ Hokkaido University Hospital, Department of Cancer Center, Kita 14, Nishi 5, Kita-Ku, Sapporo Hokkaido, Japan

^2^ Yokohama Municipal Citizen's Hospital, Department of Respiratory Medicine, 1-1 Mitsuzawanishimachi, Kanagawa-ku, Yokohama, Kanagawa, Japan

^3^ Osaka City General Hospital, Department of Medical Oncology, 2-13-22 Miyakojima-hondori, Miyakojima-ku, Osaka, Japan

^4^ Hamamatsu University School of Medicine, Department of Chemotherapy, 1-20-1 Handayama, Higashi-ku, Hamamatsu city, Shizuoka, Japan

^5^ National Center for Global Health and Medicine, Department of Breast and Medical Oncology, 1-21-1 Toyama Shinjuku-ku, Tokyo, Japan

^6^ Hokkaido University Hospital, Department of Gastroenterology and Hepatology, Kita 14, Nishi 5, Kita-ku, Sapporo, Hokkaido, Japan

^7^ Kanazawa University Hospital, Department of Respiratory Medicine, 13-1 Takara-machi, Kanazawa, Ishikawa, Japan

## Corresponding author

Name: Yoshito Komatsu

Address: Kita 14, Nishi 5, Kita-ku, Sapporo, Hokkaido, Japan

Tel: +81-11-706-5657

Fax: +81-11-706-5657

E-mail: ykomatsu@ac.cyberhome.ne.jp

# **Supplementary materials**

**Supplementary Table 1** Adverse events during the pharmacokinetic evaluation period

|  | Cohort 1 (*n* = 13) | | | | Cohort 2 (*n* = 17) | | | |
| --- | --- | --- | --- | --- | --- | --- | --- | --- |
|  | Formulation A | | Formulation B | | Fasting | | Fed | |
|  | All grade | Grade ≥3 | All grade | Grade ≥3 | All grade | Grade ≥3 | All grade | Grade ≥3 |
| Any events | 5 (38.5) | 1 (7.7) | 6 (46.2) | 1 (7.7) | 7 (41.2) | 0 | 5 (29.4) | 1 (5.9) |
| Supraventricular extrasystoles | 0 | 0 | 1 (7.7) | 0 | 0 | 0 | 0 | 0 |
| Angular cheilitis | 1 (7.7) | 0 | 1 (7.7) | 0 | 0 | 0 | 0 | 0 |
| Diarrhea | 2 (15.4) | 0 | 1 (7.7) | 0 | 3 (17.6) | 0 | 5 (29.4) | 1 (5.9) |
| Abdominal discomfort | 0 | 0 | 0 | 0 | 1 (5.9) | 0 | 1 (5.9) | 0 |
| Constipation | 0 | 0 | 0 | 0 | 1 (5.9) | 0 | 0 | 0 |
| Stomatitis | 0 | 0 | 0 | 0 | 1 (5.9) | 0 | 0 | 0 |
| Pyrexia | 1 (7.7) | 0 | 0 | 0 | 0 | 0 | 0 | 0 |
| Alanine aminotransferase increased | 0 | 0 | 1 (7.7) | 0 | 0 | 0 | 0 | 0 |
| Gamma-glutamyltransferase increased | 0 | 0 | 1 (7.7) | 1 (7.7) | 1 (5.9) | 0 | 1 (5.9) | 0 |
| Hemoglobin decreased | 1 (7.7) | 0 | 1 (7.7) | 0 | 0 | 0 | 0 | 0 |
| Blood alkaline phosphatase increased | 0 | 0 | 0 | 0 | 1 (5.9) | 0 | 0 | 0 |
| Weight decreased | 0 | 0 | 1 (7.7) | 0 | 0 | 0 | 0 | 0 |
| Hypoglycemia | 0 | 0 | 1 (7.7) | 0 | 0 | 0 | 0 | 0 |
| Hypokalemia | 0 | 0 | 1 (7.7) | 0 | 0 | 0 | 0 | 0 |
| Hyponatremia | 0 | 0 | 1 (7.7) | 0 | 0 | 0 | 0 | 0 |
| Decreased appetite | 0 | 0 | 0 | 0 | 1 (5.9) | 0 | 0 | 0 |
| Tumor pain | 1 (7.7) | 1 (7.7) | 0 | 0 | 0 | 0 | 0 | 0 |
| Insomnia | 1 (7.7) | 0 | 2 (15.4) | 0 | 0 | 0 | 0 | 0 |
| Proteinuria | 0 | 0 | 1 (7.7) | 0 | 0 | 0 | 0 | 0 |
| Rhinorrhea | 0 | 0 | 1 (7.7) | 0 | 0 | 0 | 0 | 0 |

Data are *n* (%)

**Supplementary Fig. 1** Study design overview

F, formulation; PK, pharmacokinetic

**
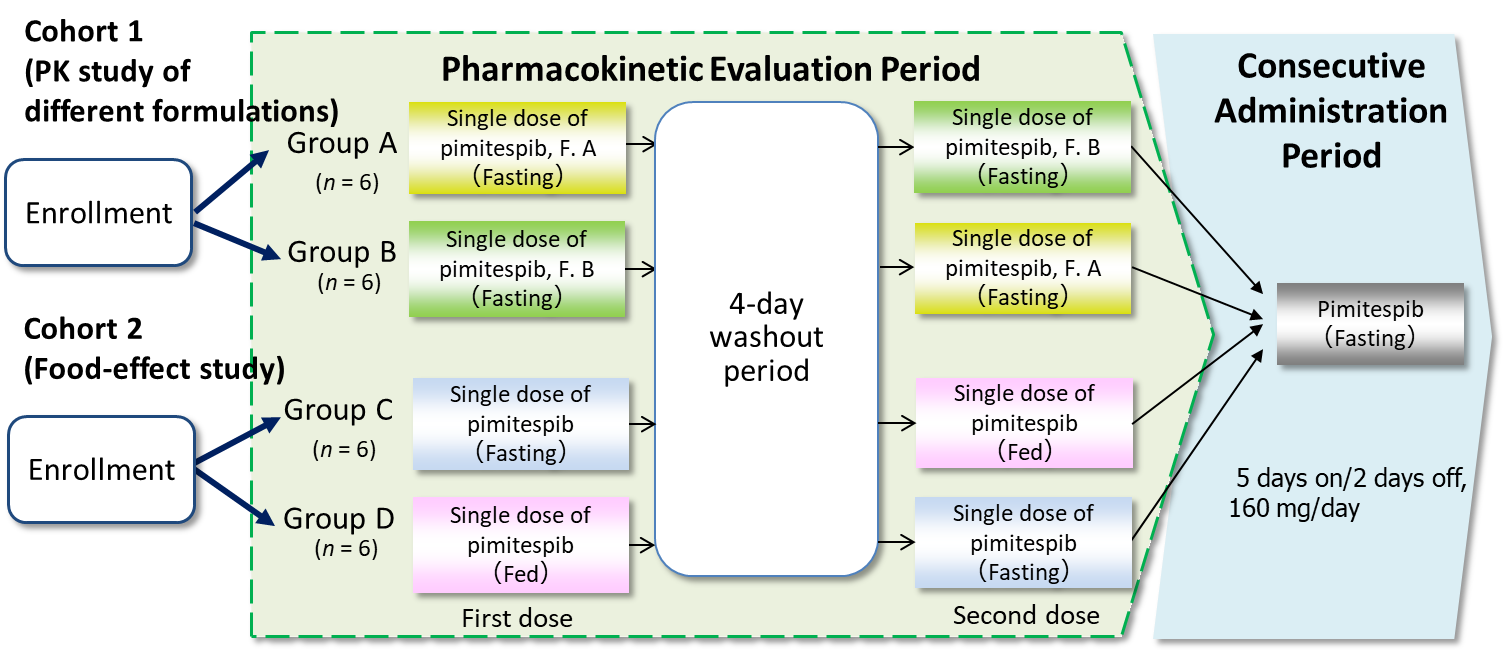
**

**Supplementary Fig. 2** Patient disposition

PK*,* pharmacokinetic


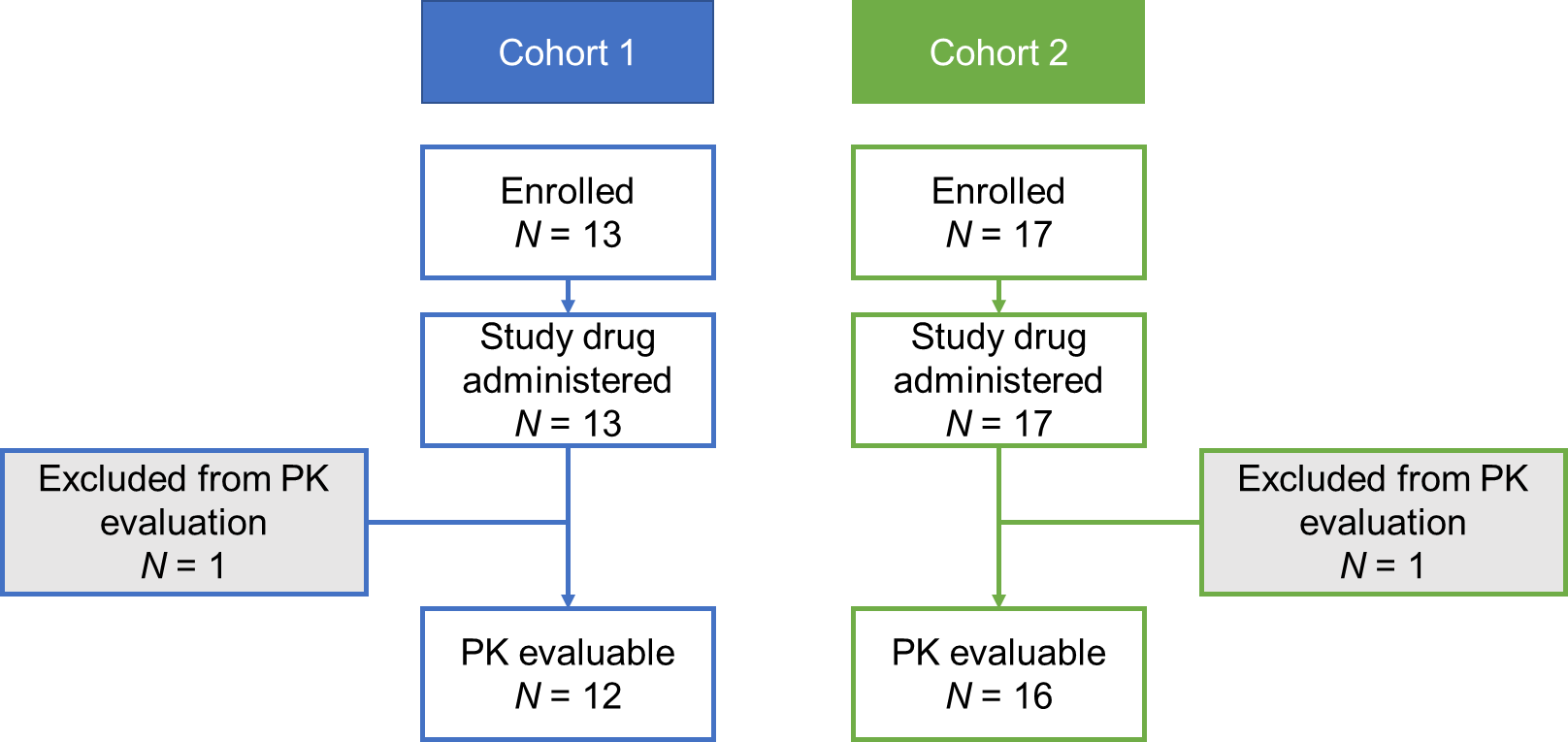

Supplement: Supplementary file 1 — Supplementary file1 (DOCX 251 KB) [file 10637_2022_1285_MOESM1_ESM.docx]
